# Likelihood of acute coronary syndrome in emergency department chest pain patients varies with time of presentation

**DOI:** 10.1186/1756-0500-5-420

**Published:** 2012-08-08

**Authors:** Ulf Ekelund, Mahin Akbarzadeh, Ardavan Khoshnood, Jonas Björk, Mattias Ohlsson

**Affiliations:** 1 Department of Emergency Medicine, Skåne University Hospital, Lund, SE-221 85, Sweden; 2 Region Skåne competence centre for clinical research, Lund, Sweden; 3 Department of Theoretical Physics, Lund University, Lund, Sweden

## Abstract

**Background:**

There is a circadian and circaseptal (weekly) variation in the onset of acute coronary syndrome (ACS). The aim of this study was to elucidate whether the likelihood of ACS among emergency department (ED) chest pain patients varies with the time of presentation.

**Methods:**

All patients presenting to the Lund ED at Skåne University Hospital with chest pain or discomfort during 2006 and 2007 were retrospectively included. Age, sex, arrival time at the ED and discharge diagnose (ACS or not) were obtained from the electronic medical records.

**Results:**

There was a clear but moderate circadian variation in the likelihood of ACS among presenting chest pain patients, the likelihood between 8 and 10 am being almost twice as high as between 6 and 8 pm. This was mainly explained by a variation in the ACS likelihood in females and patients under 65 years, with no significant variation in males and patients over 65 years. There was no significant circaseptal variation in the ACS likelihood.

**Conclusions:**

Our results indicate that there is a circadian variation in the likelihood of ACS among ED chest pain patients, and suggest that physicians should consider the time of presentation to the ED when determining the likelihood of ACS.

## Background

Despite tremendous progress in the treatment of acute coronary syndrome (ACS; i.e. unstable angina, UA, or acute myocardial infarction, AMI), our ability to diagnose or rule out ACS in emergency department (ED) chest pain patients remains relatively poor. “Rule-out” admissions to inhospital care are common, and some 7 of 10 patients admitted with a suspicion of ACS prove not to have it [1,2]. Because of the diagnostic uncertainty, ED management of patients with suspected ACS depends on the likelihood of ACS. When assessing the ACS likelihood, the ED physician considers a number of diagnostic factors, often with a Bayesian approach [3] using the likelihood ratio (LR) for each test [4-7]. For instance, the positive LR of a new ST elevation on the ECG has been reported to be 5.7-53.9, a new ST depression 3.0-5.2, pain radiating to both arms or shoulders 4.1, to the left arm 2.3, excertional chest pain 2.5, a history of myocardial infarction 1.5-3.0, diaphoresis 2.0, chest pain as the most important symptom 2.0, nausea or vomiting 1.9, positional chest pain 0.3 and pleuritic chest pain 0.2 [4-7]. To improve management, new diagnostic methods and blood tests have been suggested [8], as well as a large number of decision support models to predict the likelihood of ACS, e.g. [9-12]. In general however, routine evaluation of ED chest pain patients has not improved in later years.

It is well known that there are circadian, circaseptal (weekly) and seasonal variations in the onset of ACS [13-17], and even a circadian variation in AMI size [18]. A circadian variation of the ACS probability in chest pain patients arriving at the ED may therefore be present but has not been demonstrated. Accordingly, there is currently no basis for ED physicians to use the time of presentation to the ED as a factor when determining the likelihood of ACS, and no decision support model so far includes the time of presentation.

The aim of this study was to explore the impact of the time of arrival at the ED and the weekday on the likelihood of ACS in ED chest pain patients.

## Methods

### Setting

The Lund ED at Skåne University Hospital has a catchment area of some 300,000 inhabitants and receives a total of 65,000 patients per year with problems related to Internal Medicine, Neurology, Surgery, Orthopedics and Urology. In the hospital, there is a cardiac intensive care unit with 19 beds and an intermediate care ward with ECG monitoring for cardiac patients at 19 beds. Percutaneous coronary intervention and coronary bypass surgery are available 24 hours/day. During the patient inclusion period, there was no systematic diagnostic protocol for patients with suspected ACS, and no dedicated chest pain unit.

### Patients and diagnoses

We retrospectively included all patients presenting with non-traumatic chest pain or discomfort as chief complaint, as identified by the triage nurse and/or the responsible physician, at the Lund ED from January 1^st^ 2006 to December 31^st^ 2007. Data for each patient regarding age and sex, arrival time at the ED, admission to in-hospital care or not, and discharge diagnosis (ACS or not) were retrieved from the electronic patient records.

During the study period, diagnostic criteria for ACS (acute myocardial infarction or unstable angina pectoris) at the hospital were those recommended by the European Society of Cardiology, the American College of Cardiology and the American Heart Association [19-21]. Discharge diagnoses were made by the attending physician at the ED, or, after hospital admission, by the responsible specialist physician at the ward. No follow-up of the patients sent home from the ED was performed. The data used in the analysis were those used in the actual clinical care of the patients, and they are therefore likely to represent the routine care situation.

### Statistical analysis

We used SPSS for Windows version 18 (IBM Corp.) for the statistical analyses. The circadian and circaseptal variation in the probability of ACS was modeled using multiple logistic regression with time (12 different groups) and weekday as categorical independent variables. Sex and age (9 groups) were also included in the regression models to control for potential differences in sex and age distribution in the patient inflow at different times of the day and at different days of the week. The analyses of circadian and circaseptal variation were conducted for the entire data set, but also stratified for sex and for age (below/at least 65 years old).

### Ethical approval

This study was approved (dnr 384/2007) by the Regional Ethics Committee at Lund.

## Results

**Figure 1:**
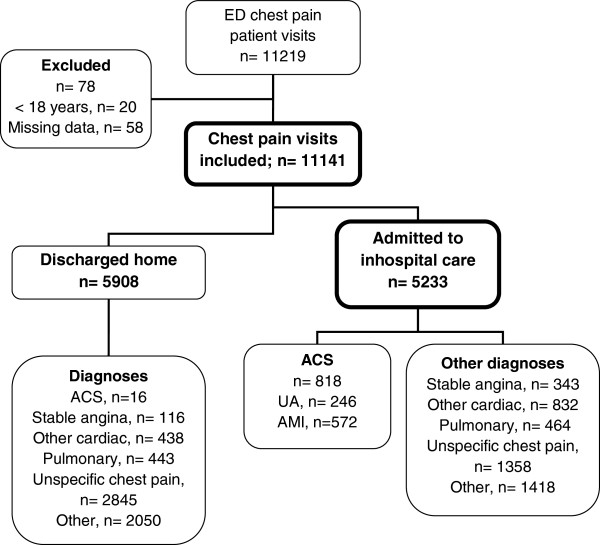
Patient flow chart.

**Figure 2:**
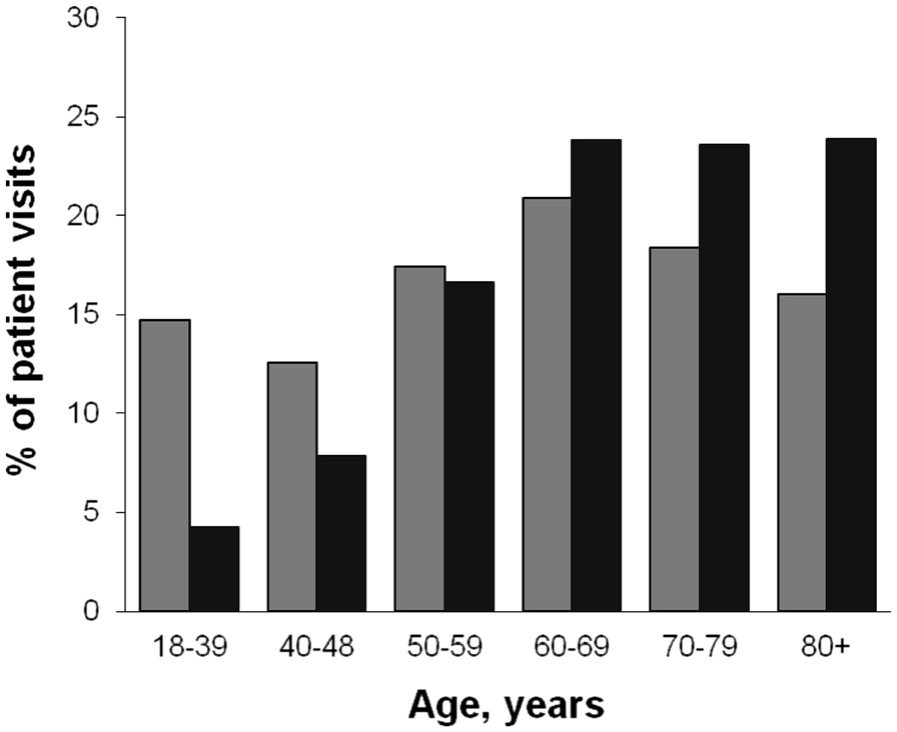
**Age distribution among all included patients, (*gray square*) and patients admitted for in-hospital care (*black square*).**

As can be seen in Figure 1, 11219 ED patient visits with acute chest pain or discomfort were identified during 2006 and 2007. Seventy-eight visits were excluded due to age < 18 years or missing data, leaving 11141 visits by 8763 patients as the study sample. Figure 2 depicts the age distribution for all patient visits and for those admitted to in-hospital care. In Table 1, the ACS likelihoods for the studied patient subgroups are shown. Among the ACS cases, 35% were women and 67% were ≥ 65 years.

**Table 1:** Number, age and ACS likelihood among presenting and admitted study cases

	**Mean age years (SD**^**a**^**)**	**ED visits (n)**	**Admitted cases (n)**	**ACS likelihood (prevalence)**	
				**Cases presenting to the ED**	**Admitted cases**
All patient visits	60 (18)	11141	5233	7.5%	16%
Men	59 (18)	5789	2979	9%	18%
Women	62 (19)	5352	2254	6%	13%
Patients < 65 years	47 (13)	6263	2152	4.5%	13%
Patients ≥ 65 years	77 (7.8)	4878	3081	11%	18%

^a^Standard deviation.

### Patients presenting to the ED

**Figure 3:**
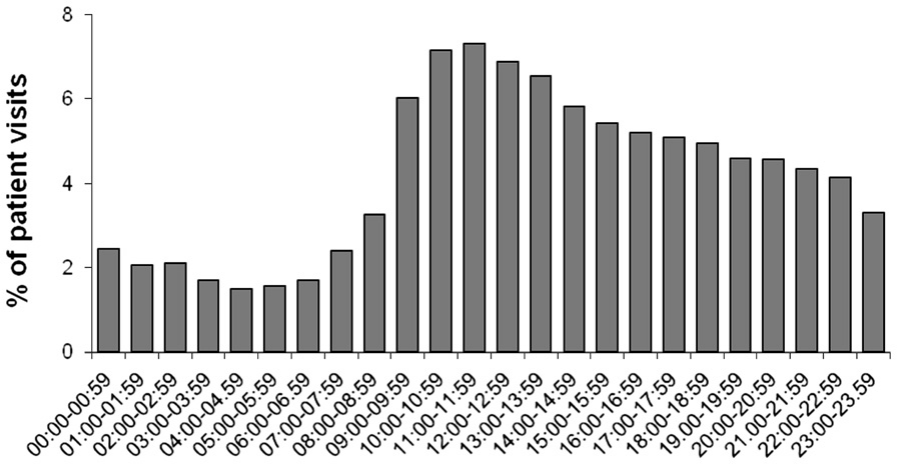
Circadian inflow of chest pain patients to the ED.

**Figure 4:**
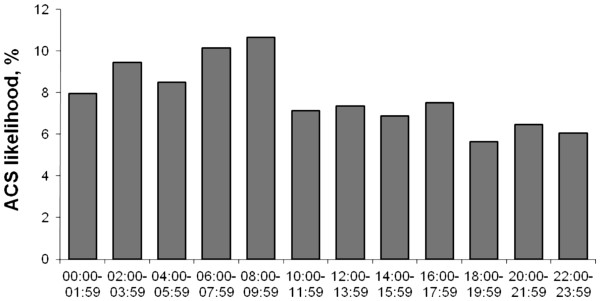
Circadian variation in ACS likelihood among chest pain patients presenting to the ED.

As can be seen in Figure 3, the peak ED inflow of chest pain patients was between 10 am and noon. This pattern was similar for men and women, and for patients under and over 65 years. The inflow of ACS patients showed a similar pattern but was highest already at 9 to 10 am (data not shown). The likelihood of ACS among presenting chest pain patients showed a clear but moderate circadian variation (Figure 4; p = 0.01 in the multiple logistic regression model), with a peak 8–10 am (10.6%) and a nadir at 6–8 pm (5.6%). This diurnal variation was mainly explained by a variation in the ACS likelihood for female (p = 0.006) and younger patients (< 65 years; p = 0.02), with no significant variation in male (p = 0.07) and older patients (p = 0.26). In women, the highest and lowest likelihoods were 9% and 2.5%.

The circaseptal variation of chest pain patient inflow was modest with Monday as the busiest day (18% of weekly patients) and Friday as the least busy day (12%). There was no significant circaseptal variation in the likelihood of ACS (p>0.3).

### Patients admitted to in-hospital care

Among chest pain patients admitted from the ED to inhospital care (n = 5233), the average ACS likelihood was higher for men than for women, and also higher for older than for younger patients (Table 1). The likelihood of ACS among admitted cases was 20% for those arriving at the ED at 8–10 am, and 13% at 6–8 pm, but the circadian variation was not statistically significant (p = 0.06).

## Discussion

This study indicates that there is a diurnal variation in the likelihood of ACS among chest pain patients presenting at the ED. To our knowledge, this variation has not been described before. The diurnal variation was more marked among females and younger patients, and was not explained by differences in sex and age distribution in the patient inflow at different times of the day.

The circadian variation in the ACS likelihood was clear but moderate, with a higher likelihood midnight-10 am (Figure 4), a peak at 6–10 am and a nadir at 6–8 pm. The obvious reason for this likelihood variation was the different diurnal inflows of chest pain patients with and without ACS. The causes of the different inflows of these groups are unclear, but there are at least two possible explanations. The first is the well established circadian variation of ACS symptom onset [13-17], which would cause ACS patients to present at the ED primarily in the morning. The second is that patients with milder chest pain and “harmless” diagnoses may hesitate to go to the ED during the inconvenient hours between midnight and 10 am. Other possible causes include different prehospital delays in the two groups owing to different symptom histories, comorbidities and/or availabilities of transportation (e.g. ambulance vs public transportation). Whatever the cause, the present data indicate that time of presentation to the ED is a diagnostic factor when determining the likelihood of ACS in ED chest pain patients.

The ED physician often determines the ACS likelihood using Bayesian reasoning [3] and diagnostic information mainly from the symptom history, the ECG and blood markers of myocardial injury, together with factors such as age, sex and previous diseases [4-7,20]. In the present study, the odds ratio for ACS for a patient presenting 8–10 am as compared to 6–8 pm was 2.0. This odds ratio is in the same order of magnitude as the impact on the odds (i.e. LR) for ACS of pain radiating to the left arm (LR 2.3), diaphoresis (LR 2.0), a history of myocardial infarction (LR 1.5-3.0), and chest pain as the most important symptom (LR 2.0), and larger than the impact of male sex in the present study (odds ratio 1.6; see Table 1). Time of presentation to the ED may thus be as important as many well-established diagnostic factors when assessing the likelihood of ACS.

The demonstration of a diurnal variation of the ACS likelihood may have at least two clinical implications. First, knowledge of this variation might influence the ED physician’s decision-making. Patients presenting at 9 am had almost twice the ACS risk of those presenting at 7 pm (Figure 4), and in female patients, the risk was 3 times higher. Since the likelihood variation must still be viewed as modest however, clinicians may take the time of arrival into account mainly when ECG, blood markers and symptoms are inconclusive, like they do with other clinical information with a modest LR such as gender, diaphoresis or a previous MI [5,7]. Second, mathematical decision support models for the prediction of ACS may be improved by the addition of a factor related to the time of presentation. Published models so far have included e.g. the ECG, previous coronary artery disease, patient age, pain duration, localization and similarity to previous angina or AMI, diabetes and sex [10-12,22,23]. Currently, these decision support models are rarely used in routine care, but if significantly improved by a time factor, their clinical use may increase.

The present inflow pattern of chest pain patients was very similar to the overall patient inflow at our and other [24-26] EDs, with a noon peak and an early morning nadir. Further, the inflow pattern of ACS patients was similar to that reported for AMI patients in the UK [27].

In patients with acute myocardial infarction (AMI), the average delay from symptom onset to presentation at the hospital is 2–4 h [28]. If the delay was similar for the ACS patients in the present study, the incidence of ACS symptom onset was highest 6–8 am. This is somewhat earlier than in previous studies, where the peak incidence of AMI symptom onset was at 8–10 am [17,29-31]. However, the average delay may be different depending on society resources, health care organization and patient population [28], and may well be different in the present study which included ACS cases than in previous studies with only AMI cases.

### Study limitations

This study was performed in a single university hospital ED and did not include data on chest pain duration or characteristics, prehospital delay or comorbidities in the patients. Although the diurnal presentation pattern and the age and sex distribution suggest that our patients were similar to those in other EDs [24-27], the present results need to be confirmed in other patient populations and health care settings. No follow-up was performed of the patients sent home from the ED, and a small fraction of the ACS cases might therefore have been missed. Since this is probably rare at our ED [32], it is unlikely that this would have influenced the results significantly.

## Conclusions

These results indicate that there is a circadian variation in the likelihood of ACS among chest pain patients presenting to the ED. The causes of the variation are likely multiple, and probably include the well-known diurnal variation in ACS symptom onset. The results suggest that physicians should consider the time of arrival at the ED when assessing the likelihood of ACS in ED chest pain patients, but additional studies are needed to confirm this in other populations and health care settings.

## Competing interests

The authors declare that they have no competing interests.

## Authors' contributions

UE conceived and designed the study, interpreted the data and wrote the manuscript. MA and AK collected and interpreted the data and critically revised the manuscript. JB provided expert statistical advice in the design and presentation in the study and critically revised the manuscript. MO collected and organized the electronic data, and critically revised the manuscript. All authors read and approved the final manuscript.
